# Maternal and paternal perinatal depressive symptoms associate with 2- and 3-year-old children’s behaviour: findings from the APrON longitudinal study

**DOI:** 10.1186/s12887-019-1775-1

**Published:** 2019-11-13

**Authors:** Nicole Letourneau, Brenda Leung, Henry Ntanda, Deborah Dewey, Andrea J. Deane, Gerald F. Giesbrecht, The APrON Team

**Affiliations:** 1 Faculty of Nursing and Cumming School of Medicine, Departments of Pediatrics, Psychiatry, & Community Health Sciences, University of Calgary, Calgary, AB T2N 1N4 Canada; 2 Owerko Centre at the Alberta Children’s Hospital Research Institute, University of Calgary, Calgary, AB T2N 1N4 Canada; 3 Cumming School of Medicine, Department of Community Health Sciences, University of Calgary, Calgary, AB T2N 1N4 Canada; 4 Cumming School of Medicine, Department of Pediatrics, University of Calgary, Calgary, AB T2N 1N4 Canada; 5 Cumming School of Medicine, Departments of Pediatrics & Community Health Sciences, University of Calgary, Calgary, AB T2N 1N4 Canada; 6 Cumming School of Medicine, Departments of Pediatrics, Psychology, & Community Health Sciences, University of Calgary, Calgary, AB T2N 1N4 Canada

**Keywords:** Perinatal depression, Prenatal depression, Postpartum depression, Maternal, Paternal, Behavioural problems, Young children, Longitudinal

## Abstract

**Background:**

Prenatal and postnatal depressive symptoms are common in expectant and new mothers and fathers. This study examined the association between four patterns of probable perinatal depression (mother depressed, father depressed, both depressed, neither depressed) in co-parenting mothers and fathers and their children’s internalizing and externalizing behaviours at 24 and 36 months of age. The influence of sociodemographic, risk and protective factors was also examined.

**Methods:**

Depressive symptoms were measured during pregnancy and at 3 months postpartum and children’s behaviour was assessed at 24 and 36 months of age. Families (*n* = 634) provided data on their children’s internalizing (i.e. emotionally reactive, anxious/depressed, somatic complaints, withdrawn and total) and externalizing (i.e. attention problems, aggression and total) behaviour. Marginal models were employed to determine the relationship between children’s behaviour over the two time points and the four patterns of probable parental depression. Sociodemographic variables as well as risk (stress) and protective (social support) factors were included in these models.

**Results:**

In the perinatal period 19.40% (*n* = 123) of mothers scored as probably depressed and 10.57% (*n* = 67) of fathers. In 6.31% (*n* = 40) of the participating families, both parents scored as probably depressed and in 63.72% (*n* = 404) neither parent scored as depressed. For children’s emotionally reactive, withdrawn and total internalizing behaviours, both mothers’ probable depression and mothers and fathers’ co-occurring probable depression predicted higher scores, while for children’s aggressive behaviour, attention problems, and total externalizing behaviours, only mothers’ probable depression predicted higher scores, controlling for sociodemographic, risk and protective factors.

**Conclusions:**

While probable perinatal depression in mothers predicted 2 and 3 year-old children’s behavioural problems, co-occurrence of depression in mothers and fathers had an increased association with internalizing behavioural problems, after considering sociodemographic, risk and protective factors. Health care providers are encouraged to consider the whole family in preventing and treating perinatal depression.

## Background

Affecting one in five children [1], the prevalence of internalizing (e.g. anxiety, depression) and externalizing (e.g. aggression, hyperactivity) behavioural problems in children is increasing worldwide [2]. Retrospective and prospective studies of adults with mental disorders reveal childhood origins, with 70% recalling and 50% demonstrating internalizing and externalizing behavioural problems from an early age [3–6]. In Canada, a country of 37 million people, the Mental Health Commission estimates the lifetime costs of childhood mental disorders is $51 billion per year [7]. Given the social and economic costs of childhood behavioural problems, their prevention has been recognized as a public health priority [8]. Behavioural problems in children are consistently associated with mothers’ (see review: [9]) depressive symptoms and increasingly with fathers’ depressive symptoms in the perinatal period [10, 11]. The degree to which concurrent symptoms in both parents affect young children’s behavioural problems is poorly understood and could offer direction for early public health and mental health interventions.

Depressive symptoms often occur prenatally and/or postnatally, that is, in the perinatal period. While the greatest lifetime risk for depressive symptoms for both women and men is in the first year after their child’s birth [12], symptoms are often present during the prenatal period, affecting up to 15% of pregnant women and 12% of expectant men. In the first 3 months postpartum, 15% of new mothers [13] and 8% of new fathers [14] have been found to display depressive symptoms. Depression is characterized by depressed mood, loss of interest or pleasure in daily activities, and at least three other symptoms including psychomotor agitation or retardation, insomnia or hypersomnia, reduced concentration and decisiveness, fatigue or loss of energy, suicidal ideation and mental confusion. Symptoms persisting over a 2 week period and significantly impacting daily functioning warrant a diagnosis of major depressive disorder [15]. While maternal postnatal depressive diagnosis has been observed to predict children’s behavioural problems, so have sub-clinical levels of depressive symptoms [11].

When mothers experience depressive symptoms postnatally, 24 to 50% of their partners (typically fathers) also experience symptoms [16]. In a systematic review, depressive symptoms in mothers led to increased symptoms in fathers [17]. Rates of co-occurring depression in mothers and fathers range from 2.3% at 12 weeks postpartum [18] to 5.4% measured at 2 weeks after birth [19] in low-risk samples. A large body of research demonstrates that children exposed to maternal postpartum depressive symptoms are at increased risk for poor cognitive, emotional, social, and physical outcomes [20–24]. A meta-analysis of 193 studies showed that maternal postpartum depressive symptoms predicted more internalizing and externalizing behavioural problems in children and young people ranging in age from infancy to 20 years [25]. Five-year-old children of mothers who experienced depressive symptoms in the postpartum period also demonstrated less ability to handle stress and engage with peers [26] and were more likely to have affective and anxiety disorders in early adolescence [27]. Indeed, maternal postpartum depression has been labeled a stressor due to its repeated demonstration as a risk factor to children’s behaviour and mental health [28].

The impact of fathers’ depressive symptoms on children may be equally harmful as mothers’ symptoms, but relatively less research has been conducted to support this assertion. One study of nearly 11,000 men enrolled in a large cohort study showed an association between new fathers’ depressive symptoms and behavioural and mental disorders in their children 7 years later [29], demonstrating the long lasting effect of fathers’ symptoms on children. Higher social support has long been known to protect against depressive symptoms in mothers [30], particularly when fathers are the support provider [31], and fathers have been explicitly described as buffering the impact of maternal depressive symptoms on children’s behavioural problems [32]. Fathers’ symptoms of depression have been associated with low social support from their partners, which may occur when mothers are depressed [16]. As a result, the potential harm to children could be increased when both parents suffer from depressive symptoms in the perinatal period. However, very little research has examined the impact of maternal and paternal depression—occurring concurrently in the perinatal period—on children. A study that examined the impact of postpartum, as opposed to perinatal, depression in both parents on children reported more negative child temperament at 3 months [33]. Other research on concurrent postnatal, as opposed to perinatal, depression suggests impacts may be due to additional factors. For example, when mothers’ and fathers’ depression were examined together, the influence of fathers’ postpartum depression on child behaviour at 3.5 years and 7 years of age was mediated by maternal postpartum depression and the stressful life event of couple conflict [34].

Generally, girls experience more internalizing (e.g. depression, anxiety) and fewer externalizing (e.g. aggression, hyperactivity) behavioural problems than boys [35], with symptoms often persisting into adulthood [36]. Paternal postpartum depression predicts increased externalizing behavioural problems in 3 to 5 year old boys [37] as does maternal postpartum depression, along with poorer cognitive development in children up to 5 years of age [22].

In summary, has been recommended that more studies include both mothers and fathers to better understand the impact of perinatal depression in a family on children [16, 38]. Thus, the objectives of this study were to determine: (1) the association between the four patterns of perinatal symptoms (mother depressed, father depressed, both depressed, neither depressed) in mothers and fathers and their young children’s internalizing and externalizing behaviours and (2) how the inclusion of sociodemographic (e.g. child sex, parental age), risk (i.e. maternal and paternal stress) and protective (i.e. maternal and paternal social support) factors affect the associations.

## Methods

This is a related follow-up study to a previously published paper [18] on predictors of co-occurring postpartum depressive symptoms in parents drawn from the Alberta Pregnancy Outcomes and Nutrition (APrON) study. APrON is a longitudinal community cohort study that began in early pregnancy. Families are currently being followed up at 12 years of age; however, this paper focuses on data collected prenatally and at 3 months postpartum from mothers and fathers, and at 24 and 36 months of age from the children. Additional details about APrON are published elsewhere [39]. The study received all the appropriate ethical approvals and both mothers and fathers provided written informed consent for themselves and their children.

### Sample

Eligibility criteria for APrON are reported in our previous paper on this sample [18]. In APrON, mothers are technically defined as birth or biological mothers and fathers are technically defined as fathers according to mothers and fathers' self-reports. Eligible parents for this analysis included partnered or co-parenting mothers and fathers during the perinatal period and from which data were available both prenatally and at 3 months postpartum. While married or common-law status was not an inclusion criteria, evidence of co-parenting in the perinatal period was determined by both parents completing the questionnaires. Completing APrON questionnaires represented a significant time commitment which we judged to be evidence of engagement in co-parenting. Thus, the final sample included, co-parenting mothers and fathers and their children. An additional inclusion criteria for children was that complete data were available on their behaviour at 24 and 36 months of age. Analysis revealed that the children who had complete data at time point 1 (24 months) and 2 (36 months) were not statistically significantly different from the children who were missing data at either timepoint on any of the sociodemographic study variables. Children were not excluded for any reason.

### Data collection

At enrollment in the first or second trimester, partnered/co-parenting mothers and fathers were asked similar questions about their sociodemographic (i.e. age, marital status, income, education, ethnicity, place of birth) characteristics and mothers were asked about parity (i.e. number of live-born children). At 3 months postpartum, mothers and fathers were again asked about their marital status as well as information on their infant’s biological sex, weight and gestational age at birth. Further, at 3 months postpartum, data were collected on risk and protective factors including maternal and paternal stress, using the Stressful Life Events Questionnaire (SLEQ) [40] and social support from Statistics Canada’s Social Support Survey [41]. Symptoms of depression were assessed using the Edinburgh Depression Scale (EDS) [42]. This measure was administered to mothers in the first and/or second trimester and third trimester of pregnancy and again at 3 months postpartum and to fathers in either the first or second trimester and again at 3 months postpartum. Prenatal and 3 month questionnaires were completed either in person or returned by mail. Mothers reported on their children’s behaviour using the Child Behavior Checklist (CBCL) (Achenbach & Rescorla, [43]) at 24 and 36 months of age in mailed questionnaires.

### Measures

#### Predictor

For mothers and fathers, probable perinatal depression was defined as a score above the Edinburgh Depression Scale (EDS) cut-off at any of the prenatal or postnatal measurement time points. (The term "probable" is used to reflect lack of confirmation by physician diagnosis and the use of a symptom scale to measure depression.) The EDS is a 10-item, self-report scale widely used in research and clinical screening depressive symptoms during the perinatal period [42, 44, 45]. It has acceptable reliability and test-retest reliability and correlates well with other measures of depression [46]. For women, in the prenatal period, sensitivity and specificity of the EDS are 79 and 97% for first trimester at the cut-off of > 11, 70 and 96% for second trimester with the cut-off of > 9, and 76 and 94% for third trimester with the cut-off of > 9 [44]. To estimate minor probable depression, the original developers and many other researchers recommend using at least > 9 as the cut-off score [42, 47, 48]. Thus, in our study, we used the accepted cut-off of EDS > 10 [44] and for fathers, we used a cut-off of EDS > 9, just slightly lower than for mothers, as recommended [14]. Similar to our previous paper [18], four groups were created for patterns of parents’ depression: (i) mother at or above cutoff at least one measurement time point and father below cut-off at every measurement time point (“depressed mother, non-depressed father”); (ii) father at or above cut-off at least one measurement time point and mother below cut-off at every measurement time point (“non-depressed mother, depressed father”); (iii) both father and mother above the cut-off for probable depression at any measurement time point (“both depressed”); and (iv) neither mother nor father at or above cut-offs for probable depression at any measurement time point (“neither depressed”).

#### Risk and protective factors

The Stressful Life Events Questionnaire (SLEQ) assessed whether or not parents experienced any of seven stressful life events including serious accident/illness or death of a close friend/family member, separation or divorce, serious argument with partner, physical abuse by partner or sexual abuse. The scores range from 0 to 7, with higher scores indicating more stressful life events [40]. To measure the protective factor of social support, we administered the Social Support Questionnaire, which consists of four questions addressing emotional, instrumental, informational and affirmational support [49]. Cronbach’s alpha was assessed at .80 [49] and scores range from 0 to 16, with higher scores indicating more social support.

#### Outcome

The 100-item Child Behavior Checklist Preschool (CBCL) 1 ½ -5-LDS [43] was completed by mothers at 24 and 36 months of child age to assess children’s internalizing and externalizing behavioural problems. For internalizing behaviours, scores are available for four “syndromes” (i.e. emotionally reactive, anxious/depressed, somatic complaints, and withdrawn) and for externalizing behaviours, scores are available for two syndromes (i.e. attention problems, aggressive behaviour). The syndrome scores may be summed to create an internalizing total behaviour score as well as an externalizing total behaviour score. Scores range from 0 to 10 for attention problems, 0–16 for both anxious/depressed and withdrawn behaviours, 0–18 for emotionally reactive, 0–22 for somatic complaints, and 0–38 for aggressive behaviours. Internalizing total raw scores can range from 0 to 72 and externalizing total raw scores from 0 to 48. In all cases, higher scores indicate greater problems. The CBCL has excellent convergent validity and consistency for the internalizing and externalizing total behaviour scales (α = 0.87 and 0.89, respectively). Raw scores were used in the analysis.

### Analysis

The data described above is longitudinally clustered within individuals as measurements taken within each child (i.e. at 24 and 36 months) are likely to be more correlated than measurements between children. As our research question seeks to determine the overall effect of parental depression on children’s behavior, rather than individual differences from the overall population average, we selected a marginal modeling approach [50]. This model uses the same framework as linear mixed models for estimating fixed effects and covariance parameters. The marginal model is specified as follows, using the outcome of internalizing behaviour total score as the example.

Let Internalizing total_*ij*_, represent repeated continuous measurement for children’s behavior for child *i* (*i* = 1, … 634), taken at the *j*^th^ time, where 24 and 36 months equate to *j* = 1 and 2 respectively.

- Internalizing total_ij_ = β_0_ + β_1_ depression group + β_2_ maternal age + β_3_ paternal age + β_4_
- maternal education + β_5_ paternal education + β_6_ maternal ethnicity + β_7_ paternal
- ethnicity + β_8_ maternal birth in Canada + β_9_ paternal birth in Canada + β_10_ maternal
- parity + β_11_ child biological sex + β_12_ maternal stress + β_12_ paternal stress + β_13_
- maternal social support + β_14_ paternal social support + ε_ij_

where ε_ij_ ~ N (0, Σ) for child *i* and time *j*, Σ is the variance covariance matrix for the residuals, specified as unstructured. The unstructured covariance matrix was preferred to a structured alternative as the latter may constrain the model unnecessarily. Further, the model is described in terms of random residuals ε_ij_ which are correlated because they come from the same person at time *j*, i.e., 24 and 36 months of age*.* In contrast to linear mixed models, the marginal model does not involve random effects, so inferences cannot be made about them as in mixed models [51]. The maximum likelihood (ML) framework was used in model estimation. We fitted 8 separate models including 5 for the measures of internalizing behaviours, i.e. (i) emotionally reactive, (ii) anxious/depressed, (iii) somatic complaints, (iv) withdrawn and (v) internalizing behaviour total and 3 separate models for the measures of externalizing behaviours, i.e. (vi) attention problems, (vii) aggressive behaviour, (viii) externalizing behaviour total.

The model selection procedure was sequential for each outcome which involved first fitting a model with all the covariates, then using stepwise elimination of non-significant covariates (alpha set at .05) beginning with the covariate with the highest alpha. As the interest was in understanding the associations between the depression group to which couples belonged (i.e. non-depressed couple; depressed mother, non-depressed father; non-depressed mother, depressed father; depressed couple) and the outcomes, controlling for other factors, this variable was not removed from the model if not found to be significant. In post-hoc analyses, we also tested the interaction term of depressed father with depressed mother. All models were fitted using Statistical Analysis System (SAS) Procedure (SAS 9.4). The sample characteristics were described using descriptive summaries, including frequencies, means and standard deviations as appropriate. Spearman correlations were also examined.

## Results

Sociodemographic and descriptive characteristics of the sample are listed in Table 1. The percent of the sample of mothers scoring as probably depressed was 19.4% (*n* = 123), fathers as probably depressed was 10.6% (*n* = 67), both parents scoring as probably depressed in the perinatal period was 6.3% (*n* = 40) and neither mothers nor fathers scoring as probably depressed was 63.72% (*n* = 404). At 3 months postpartum, mothers were an average age of 33.2 (SD = 3.9) years (range = 21–45) and fathers were 34.5 (SD = 4.77) years (range = 19–50). Children were 52.5% males and 47.6% females, with an average birth weight of 3379.6 (481) grams and an average gestational age of 39.1 weeks. Table 2 shows the Spearman correlations for the predictors and outcomes.

**Table 1:** Frequencies of demographic and descriptive variables of mothers and fathers

	Mothers (*n* = 634)	Fathers (*n* = 634)
Sociodemographic Characteristics		
Household Income (n (%))		
$100,000/year or more	398 (62.3)	398 (62.8)
$70,000-99,999/year	135 (21.3)	135 (21.3)
$40,000-69,999/year	69 (10.9)	69 (10.9)
< $ 39,999/year	24 (3.8)	24 (3.8)
Missing	8 (1.3)	8 (1.3)
Education (n (%))		
Post Graduate	170 (26.9)	111 (17.5)
University	318 (50.2)	277 (43.7)
Trade, Technical	99 (15.6)	152 (24.0)
High School or less	40 (6.3)	77 (12.2)
Missing	7 (1.1)	17 (2.7)
Marital Status by maternal report at 3 months postnatal		
Married/Common-Law	621 (98.0)	–
Single/Not Married	8 (1.3)	–
Missing	5 (0.8)	
# Children (n (%))		
0	380 (60.0)	
1	199 (31.4)	–
≥ 2	51 (8.1)	–
Missing	4 (0.6)	
Born in Canada (n (%))		
Yes	535 (84.4)	515 (81.3)
No	94 (14.8)	103 (16.3)
Missing	5 (0.8)	16 (2.5)
Ethnicity (n (%))		
Caucasian	564 (89.0)	535 (84.4)
Non-Caucasian	65 (10.3)	79 (12.5)
Missing	5 (0.8)	20 (3.2)
Risk Factor		
Stressful Life Events		
0	431 (68.0)	381 (60.1)
1	141 (22.2)	127 (20.0)
≥ 2	51 (8.0)	62 (9.8)
Missing	11 (1.7)	64 (10.1)
Early Pregnancy Depression		
Non depressed	533 (84.07)	541 (85.38)
Depressed	95 (14.98)	75 (11.83)
Missing	6 (0.95)	18 (2.84)
Late Pregnancy Depression		
Non depressed	549 (86.59)	–
Depressed	68 (10.73)	–
Missing	17 (2.68)	–
3 Months Postpartum Depression		
Non depressed	554 (87.38)	504 (79.50)
Depressed	68 (10.73)	58 (9.15)
Missing	12 (1.89)	72 (11.36)
Perinatal Depression (depression symptoms above cut-off prenatally and/or postnatally)	123 (19.4)	67 (10.6)
	Mean [SD]	Range
Protective Factor		
Mothers’ Postpartum Social Support	14.7 [2.1]	3–16
Fathers’ Postpartum Social Support	13.7 [3.0]	1–16
Behaviour at 24 months		
Emotionally Reactive	1.51 [1.55]	0–8
Anxiety/Depression	1.16 [1.35]	0–7
Somatic Complaints	1.38 [1.46]	0–9
Withdrawn	0.77 [1.06]	0–7
Internalizing Total	4.8 [3.9]	0–25
Attention Problems	1.96 [1.67]	0–9
Aggressive Behaviour	7.71 [5.48]	0–32
Externalizing Total	9.7 [6.7]	0–41
Behaviour at 36 months		
Emotionally Reactive	1.89 [1.67]	0–8
Anxiety/Depression	1.26 [1.40]	0–7
Somatic Complaints	1.56 [1.62]	0–12
Withdrawn	1.08 [1.17]	0–7
Internalizing Total	5.8 [4.2]	0–20
Attention Problems	1.85 [1.67]	0–8
Aggressive Behaviour	7.88 [5.24]	0–29
Externalizing Total	9.7 [6.3]	0–31

**Table 2:** Spearman correlations among predictors and outcomes

Variables	1	2	3	4	5	6	7	8	9	10	11	12	13	14	15	16	17	18	19	20	21
1. Household Income	–																				
2. Mothers’ Education	0.21*	–																			
3. Paternal Age	0.14*	0.04	–																		
4. Maternal age	0.10*	0.06	0.69*	–																	
5. Paternal Ethnicity	0.07*	−0.03	−0.09*	0.00	–																
6. Maternal Ethnicity	0.06*	0.02	−0.08*	−0.07*	0.44*	–															
7. Child Biological Sex	−0.02	0.02	0.02	0.08*	−0.06*	− 0.05	–														
8. Number of children	− 0.00	− 0.03	0.24*	0.29*	0.06*	0.02	0.01	–													
9. Depression	0.14*	0.09*	−0.07*	− 0.08	0.06*	0.02	0.01	−0.06*	–												
10. Mothers’ Social Support	0.10*	0.05	−0.09*	−0.13*	0.08*	0.08*	−0.06*	−0.17*	0.25*	–											
11. Fathers’ Social Support	0.02	0.00	−0.07*	−0.12*	0.03	0.08*	−0.06	−0.13*	0.15*	0.32*	–										
12. Fathers’ Stress	−0.02	0.02	0.05	0.02	−0.04	−0.03	0.07*	0.10*	−0.20	−0.20*	− 0.18*	–									
13. Mothers’ Stress	−0.11*	0.01	0.03	0.05	0.03	−0.01	0.03	0.05	−0.15*	−0.24*	− 0.09*	0.56*	–								
14. Emotionally reactive	−0.07*	−0.01	−0.13*	0.06*	0.05	0.03	−0.08*	−0.10*	− 0.12*	−0.03	− 0.00	0.05	0.09*	–							
15. Anxious/Depressed	−0.03	−0.09*	−0.02	0.01	−0.05*	− 0.08*	−0.11*	0.03	−0.05*	− 0.03	−0.07*	0.06*	0.06*	0.42*	–						
16. Somatic Complaints	−0.03	−0.07*	−0.08*	− 0.11*	−0.09*	− 0.04	−0.03	− 0.06*	−0.08*	− 0.09*	−0.07*	0.06*	0.01	0.28*	0.27*	–					
17. Withdrawn	−0.10*	−0.09*	−0.10*	− 0.04	−0.01	− 0.09*	−0.04	− 0.13*	−0.06*	− 0.05	−0.00	0.05	0.05	0.38*	0.36*	0.25*	–				
18. Attention problems	−0.08*	− 0.12*	− 0.11*	− 0.13*	−0.02	− 0.07*	0.03	− 0.03	−0.10*	− 0.12*	−0.05	0.08*	0.07*	0.42*	0.26*	0.24*	0.32*	–			
19. Aggressive Behaviour	−0.13*	−0.07*	−0.08*	− 0.03	0.03	− 0.00	0.02	0.01	−0.13*	− 0.11*	− 0.07*	0.09*	0.11*	0.57*	0.36*	0.32*	0.38*	0.57*	–		
20. Total Externalizing Behaviour	−0.13	−0.10*	− 0.09*	− 0.06*	0.02	− 0.01	0.03	0.00	−0.15*	− 0.11*	−0.04	0.09*	0.12*	0.58*	0.36*	0.33*	0.40*	0.73*	0.97*	–	
21. Total Internalizing Behaviour	−0.08*	−0.09*	−0.11*	− 0.08*	−0.03	− 0.05	−0.09*	− 0.06*	−0.16*	− 0.08*	−0.04	0.10*	0.08*	0.75*	0.69*	0.66*	0.64*	0.45*	0.59*	0.60*	–

*p-value < 0.05

### Internalizing Behaviours

For emotionally reactive behaviours, both mothers’ probable depression and mothers and fathers’ co-occurring probable depression, along with being a female child, mothers’ lower parity and mothers’ higher stress significantly predicted higher scores. For anxious/depressed behaviours, mothers’ probable depression, being a female child, having a mother with less than a university education, and mothers being non-Caucasian significantly predicted higher scores. For somatic complaints, only mothers’ probable depression and having a mother with less than a university education and mothers’ lower social support significantly predicted higher scores. For withdrawn behaviours, probable co-occurring depression, being a female child, mothers’ lower parity and income, mothers being non-Caucasian, and lower maternal social support significantly predicted higher scores. Finally, for total internalizing scores, both mothers’ and co-occurring probable depression, along with being a female child, lower mothers’ education, mothers’ lower parity, having a non-Caucasian mother, and mothers’ stress significantly predicted higher scores. See Table 3.

**Table 3:** Predictors of Children’s internalizing behaviours^a^

	Emotionally reactive	Anxious/depressed	Somatic complaints	Withdrawn	Total internalizing
Effect	Estimate (SE)	Estimate (SE)	Estimate (SE)	Estimate (SE)	Estimate (SE)
Intercept	1.69 (0.15)	0.88 (0.08) *P* = < 0.000	2.28 (0.39) *P* = < 0.000	1.56 (0.31) *P* = < 0.000	4.71 (0.18) (*p* < 0.001)
Groups (ref: Non- Depressed Mothers and Fathers)					
• Depressed Fathers	−0.14 (0.18) *P* = 0.378	−0.10 (0.15) *P* = 0.494	− 0.26 (0.17) *P* = 0.134	−0.21 (0.12) *P* = 0.090	− 0.74 (0.46) (*p* = 0.111)
• Depressed Mothers	0.64 (0.14) *P* = < 0.000	0.28 (0.11) *P* = 0.019	0.30 (0.14) *P* = 0.028	0.09 (0.10) *P* = 0.334	1.47 (0.36) (*p* < 0.001)
• Depressed Mothers and Fathers	0.50 (0.23) *P* = 0.031	0.31 (0.19) *P* = 0.108	0.37 (0.22) *P* = 0.088	0.44 (0.16) *P* = 0.005	1.65 (0.59) (*p* = 0.005)
Gender: Female (ref: male)	0.32 (0.11) *P* = 0.004	0.32 (0.09) *P* = 0.001		0.16 (0.07) *P* = 0.031	0.97 (0.28) *P* = 0.001
Mother’s Household Income (ref: $70,000 or more					
Less than $70,000				0.26 (0.10) *P* = 0.013	
Mothers’ Education (ref: University Degree or more)					
• Less than Degree		0.28 (0.11) *P* = 0.009	0.37 (0.12) *P* = 0.003		0.87 (0.33) *P* = 0.009
Number of Children	−0.25 (0.08) *P* = 0.004			−0.16 (0.06) *P* = 0.006	−0.52 (0.22) *P* = 0.016
Mothers’ Ethnicity (ref: Caucasian)					
• Non-Caucasian		0.39 (0.15) *P* = 0.008		0.41 (0.12) *P* = 0.001	0.94 (0.46) *P* = 0.040
• Mothers’ Stress	0.16 (0.08) *P* = 0.040				0.40 (0.20) *P* = 0.044
• Maternal social support			−0.06 (0.03) *P* = 0.010	−0.04 (0.02) *P* = 0.025	

^a^To determine the association between the four patterns of perinatal symptoms (mother depressed, father depression, both depressed, neither depressed) in mothers and fathers and their young children’s internalizing behaviours. Marginal model standardized beta coefficients are interpreted the same way as in linear regression

### Externalizing Behaviours

For aggressive behaviour, mothers’ probable depression along with lower mothers’ education and income, mothers’ birth in Canada, and higher mothers’ stress predicted higher scores. For attention problems, mothers’ probable depression along with lower mothers’ age, lower education and lower mothers’ social support predicted higher scores. Finally, for total externalizing scores, only mothers’ probable depression, along with lower mothers’ education and income and higher stress, predicted higher scores. See Table 4.

**Table 4:** Predictors of Children’s externalizing behaviours^a^

	Attention problems	Aggressive behaviour	Total externalizing
Effect	Estimate (SE)	Estimate (SE)	Estimate (SE)
Intercept	5.45 (0.81) P = < 0.000	6.62 (0.63) P = < 0.000	8.19 (0.33) P = < 0.000
Groups (ref: Non- Depressed Mothers & Fathers)			
• Depressed Fathers	0.17 (0.21) *P* = 0.413	− 0.34 (0.62) *P* = 0.580	−0.26 (0.76) *P* = 0.736
• Depressed Mothers	0.51 (0.18) P = 0.005	1.52 (0.49) *P* = 0.002	2.20 (0.60) *P* = 0.000
• Depressed Mothers and Fathers	0.13 (0.29) *P* = 0.659	1.13 (0.79) *P* = 0.156	1.41 (0.97) *P* = 0.145
Maternal Age	−0.06 (0.02) *P* = 0.000		
Mothers’ Education (ref: University Degree or more)			
• Less than degree	0.41 (0.16) *P* = 0.009		1.35 (0.56) *P* = 0.018
Fathers’ Education (ref: University Degree or more)			
Less than degree		1.34 (0.40) *P* = 0.001	
Mothers’ Household Income (ref: $70,000 or more)			
• Less than $70 k		1.23 (0.54) *P* = 0.023	1.51 (0.66) *P* = 0.024
Mother born in Canada (ref: No)			
Yes		1.14 (0.53) *P* = 0.032	
Mothers’ Stress		0.89 (0.27) *P* = 0.001	0.95 (0.33) *P* = 0.004
Maternal Social Support	−0.12 (0.03) *P* = 0.001		

^a^To determine the association between the four patterns of perinatal symptoms (mother depressed, father depression, both depressed, neither depressed) in mothers and fathers and their young children’s externalizing behaviours. Marginal model standardized beta coefficients are interpreted the same way as in linear regression

While the categorical variable for probable depression in parents was associated with many behavioural outcomes, the post-hoc analysis of the interaction term for mothers’ and fathers’ depression did not produce significant associations in any model.

## Discussion

The impact of maternal perinatal depression on children’s internalizing and externalizing behaviour at 2 years [52], 3 years [9, 53], 6 years [54] and 7 years of age [55] has been demonstrated. Research has also explored the impact of fathers’ prenatal and postnatal symptoms of depression on children’s behaviour [37]. This study may be the first to examine associations between maternal, paternal and co-occurring probable perinatal depression on behaviour in children 2 and 3 years of age in a low-risk community sample, compared to non-depressed parents, controlling for known covariates.

### Associations between probable perinatal depression and Children’s behaviour

For all the internalizing behaviours measured, significant negative associations were observed with mothers’ probable depression in the perinatal period. When probable depression co-occurred in mothers and fathers, significant negative associations were observed with only children’s emotional reactivity, withdrawn behaviours and total internalizing behaviours. Examination of the beta coefficients suggest the greatest impact was observed on total internalizing behaviours when both parents were probably depressed. In contrast, only mothers’ probable depression predicted externalizing behaviours. In general, fathers’ probable depression did not associate with children’s behavioural problems, unless mothers were also symptomatic. These associations remained significant even after accounting for sociodemographic, risk and protective factors.

The impact of co-occurring depression on young children’s behaviour is supported partially by the work of Dietz et al. [56]. In a study of 101 families, Dietz et al. found that maternal postnatal depression was significantly associated with toddlers’ externalizing and internalizing behavior problems, but only when paternal postnatal psychopathology was present. While our study was limited to the study of probable depression, not other psychopathologies, and included symptoms in the greater perinatal period, we found that co-occurring probable parental depression predicted children’s internalizing, but not externalizing behavioural problems to a greater degree than probable maternal depression only. Nonetheless, probable maternal perinatal depression independently predicted both internalizing and externalizing behavioural problems in children.

In contrast, unlike Ramchandani et al.’s [37] observation of independent associations between paternal postpartum depression and children’s behaviour, we only found associations between fathers’ probable depression when mothers were also symptomatic [37]. This may be due to our consideration of perinatal as opposed to simply postpartum symptoms. A systematic review of 21 studies showed that prenatal paternal depression predicted emotional problems in 2 month-old to 7.5 year old children, and that postnatal paternal depression predicted both internalizing and externalizing problems in 2 month old to 8 year-old children [57]. Our study may be the first to examine and compare the unique and combined associations between probable perinatal depressive symptoms in *both* mothers and fathers and children’s behaviour recruited from a low-risk community sample. Other studies have examined maternal and paternal psychopathology, not limited to probable depressive symptoms, revealing that associations were stronger between maternal than paternal psychopathology and internalizing problems in children, but not externalizing problems [58].

Our findings focus on perinatal depressive symptoms in particular, showing that both internalizing and externalizing behaviours of preschool children are significantly negatively associated with their mothers’ symptoms of depression; however, depressive symptoms in both parents also negatively predicted internalizing, but not externalizing behaviours in preschoolers. It is noteworthy that fathers’ symptoms of perinatal depression, considered in contrast to the other patterns of parents’ perinatal depression, did not associate with children’s behavioural problems, unless mothers were also symptomatic. Mothers may buffer the impacts of fathers’ probable depression on children’s behaviour, much as fathers are regarded as buffering the impacts of maternal symptoms on children [32]. Prenatally, when both parents are depressed, neither parent is able to support the other’s coping, with developmental implications for stress physiology underpinning children’s behaviour [59]. Postnatally, when both parents are depressed, parents are less likely to engage in nurturant (e.g. sensitive and responsive) interactions with infants, necessary for optimal emotional regulation underpinning children’s behavioural development [11, 60, 61].

### Associations between Sociodemographic factors and Children’s behaviour

Consistent with the literature, lower maternal household income [62] and lower maternal education [63] predicted increased behavioural problems in children. Specifically, increased children’s behavioural problems were observed in both internalizing (i.e. anxious/depression, somatic complaints, and total) and externalizing (i.e. attentional problems and total) domains when mothers had less than a university education. We also found that lower fathers’ education predicted increased externalizing behavioural problems (i.e. aggression), which has been studied far less than mothers’ education. More often, the impact of fathers’ education has been studied in older children and with respect to children’s educational attainment [64, 65]. With respect to income, lower mothers’ household income significantly predicted withdrawn, aggressive and total externalizing behaviours. While these findings are consistent with research on low-income families [66], our research showed these associations with relatively higher levels of “low-income”, i.e. at less than $70,000 CDN per year. Being a younger mother is often associated with increased behavioural problems in children, but typically based on research on adolescent parents [67]. For attention problems, we observed this finding in our sample of mothers ranging in age from 21 to 45; however the beta coefficient is very small. Having more children predicted decreased internalizing behaviours (i.e. emotionally reactive, withdrawn and total), but not externalizing behaviours. This may be due to mothers’ greater sense of competence with having more children [68]. Similar to other research, we found that overall, female children were more prone to internalizing problems [69, 70], specifically, in all but the somatic complaints syndrome. In contrast to previous research, we did not find that males were more prone to externalizing problems [70, 71]. Further, being non-Caucasian predicted higher anxiety/depression, withdrawn and total internalizing behaviours in children, which may be explained by minority status, often associated with worse behavioural outcomes [72]. In contrast, children whose mothers were not born in Canada had lower aggression scores, which may be due to the healthy immigrant effect [73]. These findings suggest that immigration may have opposite influences on internalizing and externalizing behaviours.

### Associations between risk and protective factors and Children’s behaviour

As a risk factor, mothers’ stressful life events increased children’s emotional reactivity, total internalizing behaviours, as well as aggression and total externalizing behaviours in 2 to 3 year old children. Other research has demonstrated that higher perceived stress in women during pregnancy has been associated with higher odds of total behavioral problems (OR = 1.17) and more externalizing behavioral problems (OR = 1.12) in children at 2 years of age [74]. A systematic review of 23 studies reported that maternal prenatal stress (including anxiety) was associated with negative reactivity or self-regulation in children in the first 2 years of life, with small to moderate effect sizes [75]. Similar to other studies that have reported on the effectiveness of social support in preventing children’s behavioural problems, [76, 77], we found that social support was a protective factor in the context of probable perinatal depression, specifically for somatic complaints, withdrawn behaviours and attention problems.

### Future research

Future research could examine the potential protective factor of parent-child relationship qualities, and consider the role of both mothers and fathers. For mothers, studies have repeatedly demonstrated that parental nurturance mediates associations between maternal depression and child behaviour problems [11, 55, 78, 79]. Less research has examined mediation of associations between paternal depression and children’s behaviour by nurturance [80], although a growing literature supports the value of paternal nurturance for children’s development, especially in the cognitive domain [81, 82]. Indeed, fathers’ depression has been found to negatively impact father-child interactions [83, 84] and when children display more externalizing problems, fathers tend to become more involved with their children [85]. Others have found that couple conflict mediates the association between paternal depression and child behavioural and emotional outcomes [86]. Sweeney and MacBeth’s [57] review of studies of paternal perinatal depression revealed that associations with children’s outcomes are likely mediated by marital conflict and parenting behaviours. Examination of opposite influences of immigration on internalizing and externalizing behaviours is also recommended for future study.

### Strengths and limitations

There are a number of strengths associated with this study. First our identified rates of probable depression in mothers, fathers, and both mother and fathers are consistent with other research on rates of depression in these groups [13, 53, 87]. Second, this is one of the first papers to examine the relative influences of the four patterns of probable parental perinatal depression on children’s behaviour. Third, the large sample offers the opportunity to undertake robust statistical modelling as well as consider the impact of probable depression in an uncommon situation, when both mothers and fathers have probable depression. Our ability to detect this difference may be due to our large sample size. Finally, the data set was relatively complete, with very little missing data. For demographic and descriptive variables, missing data ranged from 0 to 1.73% with the exception being fathers’ stress, with 10.1%. Results associated with this variable could be interpreted with more caution.

One issue that limits the conclusions that can be drawn from this paper is that the sociodemographic characteristics of the APrON cohort tend towards higher education and higher income; thus, findings are appropriately generalizable to similar, higher income, higher education families. Second, the self-report EDS does not diagnose depression, thus our findings are more likely generalizable to low-risk, non-clinical samples. Third, there was no prerequisite for mothers to be in remission of probable depression to report on child behavior. Accordingly, maternal report biases with regard to children’s internalizing and externalizing behaviours could have resulted. Fourth, the stability of the predictor of perinatal depression was low with 2.21% of mothers and 4.10% of fathers depressed across all time points. Thus, it is likely that some children were exposed to only depression prenatally, and others only postnatally. Fifth, the data from this community sample were necessarily skewed toward low risk of behavioural problems, resulting in very few children who scored above clinical cut-offs for the behavioural outcomes, with only 6 and 11 children in internalizing behaviours and 16 and 13 children in externalizing behaviours at 2 and 3 years respectively. Finally, while statistically significant, the pragmatic value of the findings is unclear. For example, on the measure of externalizing behaviour, possible scores range from 0 to 72. The presence of concurrent perinatal depression increased the children’s scores, on average, by 1.65 points, from 4.71 to 6.36. Thus, the clinical significance of the findings is difficult to judge.

## Conclusions

This may be the first study with a large enough sample to demonstrate that probable depression co-occurring in the perinatal period in both mothers and fathers predict internalizing behavioural problems in children, while depression in mothers independently predicted both internalizing and externalizing behavioural problems in preschool children. Further, mothers and fathers need only experience probable depression at some point during the perinatal period, not necessarily concurrently, to produce negative associations with children’s behaviour. Findings point to areas for assessment and intervention to promote the mental health of childbearing families. In particular, we recommend that fathers undergo a mental health assessment when mothers experience depressive symptoms in the perinatal period. As suggested by others [31, 88, 89], both mothers’ and fathers’ mental health ought to be assessed in the perinatal period. Health care providers are encouraged to consider the whole family in preventing and treating depression throughout the perinatal period. Future research and clinical work may be served by considering additional protective factors such as quality of parent-child relationships and parent conflict in protecting children’s behavioural development.

## Data Availability

Not applicable.

## Abbreviations

AIC: Akaike information criterion
APrON: Alberta pregnancy outcomes and nutrition
CBCL: Childhood behavior checklist
EDS: Edinburgh depression scale
SLEQ: Stressful life events questionnaire
